# Case-Informed Learning in Medical Education: A Call for Ontological Fidelity

**DOI:** 10.5334/pme.47

**Published:** 2023-04-12

**Authors:** Anna MacLeod, Victoria Luong, Paula Cameron, Sarah Burm, Simon Field, Olga Kits, Stephen Miller, Wendy A. Stewart

**Affiliations:** 1 Department of Continuing Professional Development and Medical Education, Dalhousie University, Halifax, Nova Scotia, Canada; 2 Department of Emergency Medicine, Dalhousie University, Halifax, Nova Scotia, Canada; 3 Department of Pediatrics, Dalhousie University, Halifax, Nova Scotia, Canada

## Abstract

Case-informed learning is an umbrella term we use to classify pedagogical approaches that use text-based cases for learning. Examples include Problem-Based, Case-Based, and Team-Based approaches, amongst others. We contend that the cases at the heart of case-informed learning are philosophical artefacts that reveal traditional positivist orientations of medical education and medicine, more broadly, through their centering scientific knowledge and objective fact. This positivist orientation, however, leads to an absence of the human experience of medicine in most cases.

One of the rationales for using cases is that they allow for learning in context, representing aspects of real-life medical practice in controlled environments. Cases are, therefore, a form of simulation. Yet issues of fidelity, widely discussed in the broader simulation literature, have yet to enter discussions of case-informed learning. We propose the concept of *ontological fidelity* as a way to approach ontological questions (i.e., questions regarding what we assume to be real), so that they might centre narrative and experiential elements of medicine.

Ontological fidelity can help medical educators grapple with what information should be included in a case by encouraging an exploration of the philosophical questions: What is *real*? Which (and whose) reality do we want to simulate through cases? What are the essential elements of a case that make it feel real? What is the clinical story we want to reproduce in case format? In this Eye-Opener, we explore what it would mean to create cases from a position of *ontological fidelity* and provide suggestions for how to do this in everyday medical education.

Imagine you’ve been asked to write a case for an undergraduate medical education curriculum with the direction: “Please write a case about hypertension.” That instruction sets into place a sequence of activity: you would likely go about describing the condition, its presenting clinical manifestations, relevant signs, treatment options, and prognosis. You might turn to the literature to ensure the information included is accurate, evidence-based, and up to date.

In contrast, imagine you received the direction: “Please write a case that tells the story of a person with hypertension.” The difference is subtle, but notable. The path you would take to write this case, in telling the *story* of an illness and diagnosis, brings to the fore a different focus: an attempt to convey the essence, or lived experience, of the illness. We believe this different orientation, though subtle, matters.

Text-based cases are, in fact, fundamental to medical education. They are the primary mechanism through which case-informed learning (which we will use as an umbrella term that includes the various approaches to learning with cases at their heart, including problem-based, case-based, team-based and others) occurs. We contend that the way we write and think about cases, including their format, content, and purpose, provides not-so-subtle clues about the types of information medicine takes to be real: fact, evidence, procedure.

While cases as an educational device remain surprisingly under-explored in the medical education literature, those contributions that do exist have noted an emphasis on the factual medical information rather than the way an individual’s social interactions and identity can affect a clinical encounter and health outcome [1,2,3]. This largely absent patient voice in cases, we believe, is not a product of ill-will or bad intention. On the contrary, it arises from a desire to ensure that cases are packed full of the essentials, meaning the information a student needs to move onto the next stage of their education. Herein lies the challenge: How do we determine what information is, in fact, essential to include in a case?

The missing piece here, we contend, is a philosophical issue, and more specifically, an *ontological* one. In other words, the cases we use for education reproduce unquestioned philosophical assumptions about *the nature of reality*. The philosophy of science holds that all fields, including medical education, are constituted through a set of philosophical principles, including ontology (what is real), epistemology (what we can know), methodology (how we can know), and axiology (what we value) [4]. In its simplest form, ontology can be defined as the science of being. In the world of medical education, Varpio and MacLeod [4] described ontology as what we, as a field, assume to be real.

In this manuscript, we take the position that cases are educational artefacts, and that the ontological assumptions of medicine are present (or not) in the purpose, structure, and content of the cases we use for teaching. We ask the question: what would it mean to create cases from a position of *ontological fidelity* and provide suggestions for how to do this in everyday medical education?

## Why does philosophy matter?

Medical education, along with all related fields, is steeped in philosophy. Yet, philosophical inquiry has yet to find its way to the everyday practices of our medical schools. This is despite increasing interest in integrating philosophical ideas in the academic realm, notably within *Teaching and Learning in Medicine’s* Philosophy and Medical Education series [5], *Academic Medicine*’s Philosophy of Science series [4], and the recent book *Applied Philosophy for Health Professions Education* [6]. Certainly, medical education can benefit from turning to the tools of philosophy to address medicine’s long-standing challenges through a fresh perspective [5]. As Veen and Cianciolo [5] remind us, “philosophy can be seen as the fundamental approach to pausing at times of complexity and uncertainty to ask basic questions about seemingly obvious practices so that we can see (and do) things in new ways” [5 p337].

**Figure 1:**
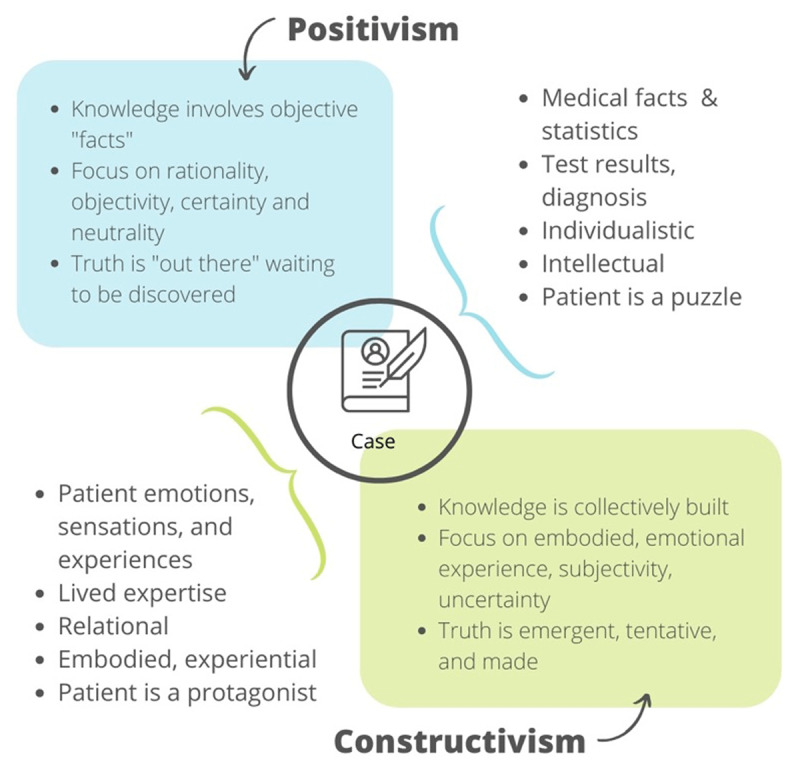
Positivist and Constructivist Approaches to Case-Based Learning.

Traditionally, medicine and medical education have embraced positivist ideals around rationality, objectivity, and neutrality [7]. Systematic practices designed to minimize uncertainty like evidence-based medicine and critical appraisal have historically maintained a position of privilege within medicine and medical education [8]. However, notions of objectivity and neutrality disguise the complex, contradictory, and often unpredictable nature of human activity [9]. In the context of research, the world of medical education has benefited from constructivist perspectives that knowledge does not exist as an objective “fact” awaiting discovery; rather, it is a social product, a testament to the subjectivities of its creators [10,11] (See Figure 1). We make room for multiple ontologies in our scholarly work, with recent contributions of critically oriented, theoretically informed social science perspectives in our journals and conferences [1,12,13]. Interestingly, however, creating the same space in our educational practices, including case-informed learning, has not been quite as successful.

## Case-Informed Learning

Case-informed learning is one of the first pedagogical approaches learners will encounter. This approach, originally conceptualized and implemented as Problem-Based Learning (PBL) by Barrows and Tamblyn in the mid 1960s [14], involves students working in small groups with a faculty facilitator or tutor to solve a clinical “problem” (i.e., a case) that simulates a real-life clinical situation [15]. The goal is to “reduce fragmentation of knowledge and acquisition of meaningless facts, to promote curiosity and teamwork, and to present a patient rather than a disease model” [16 p868]. In other words, as learners collaboratively investigate the case at hand, they develop the skills to find information and solve problems they can subsequently apply to their professional practice [15].

Case-informed learning approaches are, in fact, ontological artefacts. They can be traced to theories of rationalism, which refer to the idea that opinions and action should be based on reason and knowledge (e.g., mathematical knowledge) rather than on emotions or sensory experience, and are strongly influenced by cognitive psychology [17] as well as Dewey’s [18] encouragement of independent and experiential learning [15]. The notion of learning from a simulated case, or problem, can also be credited to Dewey, as he considered context and learning in concert with “real life” critically important.

While case-informed learning has evolved over time, with variations like case- and team-based learning gaining popularity, the narrative case story at the heart of the inquiry remains consistent. As a text-based curricular model, case-informed approaches have the potential to embrace the stories of medical practice. In reality, however, the “voice of medicine”—a technical, scientific, and professional approach—has dominated illness stories, at the expense of the patient’s “voice-of-the-life-world”: the patient’s lived experiences of events and problems in their life [19,20]. What we therefore see more often in practice are the medical-centric “hospital stories,” such as those described by Coulehan [21]:

The stories that permeate the hospital ethos don’t usually have patients as their protagonists, and often not even as ancillary characters that play human roles. Rather, patients quite frequently serve as clever or frustrating plot devices—obstacles or challenges that impair the story’s progress; or sometimes they may serve as positive plot devices, unexpected gifts that facilitate the story’s successful resolution [21 p109].

Coulehan, and other scholars of narrative [22], have encouraged us to pay attention to the work that is accomplished through stories. Narrative medicine [23,24,25] has flourished as a meaningful avenue for fostering narrative competence: “the capacity to recognize, absorb, metabolize, interpret, and be moved by stories of illness” [23 p1265]. By “(re)humanizing medicine” through portrayals of patients’ subjective experience of illness in medical education research and medical humanities curriculum [26 p113], lived experience becomes legitimized as knowledge and provides patient agency as narrator of their own story [26]. Interestingly, though, the stories that we use for teaching future physicians have received significantly less attention.

## What about the case?

Despite decades of research addressing various case-informed learning approaches, surprisingly few studies have contested the traditional format of cases [2,27,28,29]. Kenny and Beagan [30] noted that the typical construction of cases “grants ultimate authority to the voice of the doctor, excluding the voice of the patient. It constructs medical observations and interpretations as incontestable facts while devaluing patient observations as subjective and fallible” (30 p1073). In this manner, students may be accultured to view patients as problems to be solved, and themselves as problem-solvers [1].

Certainly, cases educate students about more than a particular diagnosis. The ontological (and relatedly, axiological, i.e., what we value) foundations of medicine, reinscribed through the case, implicitly teach students about what they need to concern themselves with, and what they can ignore. This, in turn, dictates not only what and how they should think, but also what and how they should feel, and how they ascribe and prioritize importance to these areas.

A key feature of case informed approaches consistently described in the medical education literature is their simulation of “real life” [3,14,17,27,31,32,33,34]. As medical educators, we must pause to ask ourselves: what type of practice do we hope this case will simulate?

## Case, Simulation, and Ontological Fidelity

Interestingly, while we recognize that case-informed learning is, in fact, a type of simulation, our field has not yet made a clear connection between cases and the concept of *fidelity*. In the context of simulation-based medical education, fidelity refers to the degree of realism we should expect, or the degree of exactness with which the simulation reproduces reality [35,36,37]. Medical educators generally focus on two types of fidelity: physical (i.e., similarity in the look and feel of the simulator) and functional (i.e., similarity in how the simulator responds to manipulation or intervention) [35,38]. Returning to our underlying philosophical assumptions, these approaches to fidelity draw on positivistic orientations, concerned with objective, measurable ways these simulations align with “real” clinical practice [37].

In another study, we brought forward the concept of *ontological fidelity* as an additional type of fidelity that merits our careful consideration [12]. In that work, we noticed that learners engaged very differently when they practiced procedures on a cadaver as opposed to a manikin because, to simplify, the cadaver was *real—*and unmistakably so. The cadaver had been a living person with a story and a history, and that former life permeated the teaching sessions [12,39]. Stated simply, then, ontological fidelity refers to the degree to which a simulator matches what a patient is: a real, human person. The ontological fidelity of cadavers was their greatest strength and inspired a very different type of practice. Despite our best efforts, no amount of technological advancement could reproduce that realness.

Extending the concept of ontological fidelity to case-informed learning, we believe that medicine, as a narrative practice, is in fact constructed through story. Certainly, there are enough true stories in medicine that we can create a compelling, and real, case—but only if we agree to engage in narrative practice, and only if we make space for the human experience of medicine in our cases.

Returning to case-informed learning, we are faced with a set of philosophical questions: What is *real*? Or, perhaps more accurately, what (and whose) reality do we want to simulate through cases? What are the essential elements of a case that make it feel real? What is the clinical story we want to reproduce in case format?

## Bringing Ontological Fidelity to Case-Informed Learning

What we currently know about case-informed learning is that cases themselves continue to be taken-for-granted in ways that reinforce the positivist ontological position that objective fact, rather than patients’ stories, emotions, and experiences should constitute the substance of the case. We propose that attending to ontological fidelity will lead to more meaningful cases. To do that, we encourage educators to pause and deliberately examine their assumptions about what is *real*, and how those assumptions translate into three elements of cases: **format, content**, and **purpose** (See Table 1).

**Table 1:** Examples of how Attending to Ontological Fidelity can Enhance the Format, Content, and Purpose of Cases in Case-Informed Learning.


		TRADITIONAL CASES	WAYS TO ENHANCE ONTOLOGICAL FIDELITY

**Format**	Description	Typical format promotes biomedical/clinical focus.	Less prescriptive format may encourage space to consider patient’s unique context and experience.

	Example	Headings include: Learning Objectives, Preparation Tasks, Resources, Case Description, Physical Exam, Laboratory Findings, Questions to Consider, Other Information	Additional/alternative headings could include: Patient Context, Practitioner’s Perspective, Illness Experience, Critical Thinking/Reflective Discussion Questions

**Content**	Description	Content includes mostly objective, decontextualized fact. The disease, rather than the patient, is the protagonist of the case.	Content integrates factual biomedical information with patient, family, and practitioner experiences. The patient is the protagonist of the case, with family members, caregivers, and healthcare workers actively contributing to the patient’s story.

	Example	Case Title: A case of leukemia Case information: symptoms at presentation, progression of symptoms, test results, interventions (e.g., medications, surgeries, etc.), broad outcome (e.g., full recovery, death) Narrative devices: The case information is presented in the passive voice (e.g., the patient was given a transfusion; counseling was provided)	Case Title: A case of a woman diagnosed with leukemia Case information: symptoms and experience of symptoms are described simultaneously; details of how tests and interventions were negotiated between practitioners and patients are provided; outcomes are placed within a specific context. Narrative devices: The story is written in the active voice, and multiple “characters” actively speak in the case.

**Purpose**	Description	The case aims to impart essential information. The case promotes succinct answers rather than active discussion.	The case integrates narrative devices and probing questions that aim to inspire rich conversation.

	Example	Questions are biomedically focused (e.g., What is a haemopoietic stem cell transplant? What are the general principles of cytotoxic chemotherapy?) The case resolves suddenly and neatly (e.g., “The patient died peacefully in her sleep, 2 years after her diagnosis.”)	Questions integrate critical questions about the patient’s experience (e.g., Why might the patient refuse treatment at this stage? How might the patient’s background influence her current experience with leukemia?) The messy experience of recovery, or death and dying during the end of life, are described realistically.


### Format

The format of text-based cases remains consistently unchallenged. While there might be variation between institutions, we have generally come to expect cases that are written concisely, and offer a traditional structure that highlights the clinical focus of the case. They generally include a description of the patient scenario where symptoms, investigations, and treatments are listed; a set of learning objectives and resources; and some guiding questions.

Although each case is unique in terms of the names and conditions of the patients represented, the framework for the case, itself, is reproduced again and again. Students can begin to find this rather boring, contributing to what has been referred to as “PBL fatigue” [40]. Additionally, these similarly structured cases present each simulated clinical encounter as more or less “the same,” detracting from the uniqueness and complexity of each patient, and deviating from the ways that spoken speech may be experienced in clinical encounters with patients and colleagues (e.g., during oral case handovers). Further, text-dense documents may pose barriers to accessibility for learners with diverse learning profiles, including those with learning differences such as dyslexia.

The reproduction of a standardized case format appears to be a long-standing practice. For example, in 1993, Good and Good observed, “No explicit attention is paid to how cases are constructed (with minimal social and personal characteristics and great physiological detail) and how sufferers are reconstructed as cases….” [41 p94].

Were we to reimagine case format to attend to ontological fidelity, we might reconsider the prescribed approach to structuring cases. While templates are undoubtedly helpful in terms of attending to all the details that need to be included, templates also serve to reinforce the expectation that all cases look the same, and relatedly, can be managed in the same way. The order of information might be changed so that each case, and the related activities it inspires/requires, is unique. Sometimes cases may feature stories from patients (which could be written or video), and in others they might include reflective comments from practitioners. Guiding questions, rather than being designed to draw out specific bits of information, might be reoriented to inspire higher level discussion, critical thinking, and empathy.

### Content

Case-informed approaches are frequently described as a “patient-centred” pedagogical approach [14,16,27,42,43,44,45,46,47]. Each case features a patient—how could they be anything else?

We believe, however, that simply having a named patient in each case does not offer the patient perspectives necessary for ontological fidelity. The patient in an educational case is often written as a narrative device: a two-dimensional vehicle for relaying biomedical or clinical information. They are presented as a list of symptoms assigned a name and, in some instances, a job. Rarely do we hear from a patient in their own voice in cases [2]. Rarely do we learn about, or even consider, the emotional elements of the case in question. Rarely do cases engage with the magnitude of the diagnosis for individual patients and what life will look like for those who have been diagnosed, particularly as they wade through lengthy weeks and months of testing and appointments, which does not equate to a paragraph or two on paper. Rarely do cases address the social realities of a diagnosis: that an illness is experienced differently depending on social location of the patient. Instead, cases are generally flat, tidy, and orderly—lacking the contours of a patient’s embodied experience, agency, and humanness. While this flatness does not arise from any ill intent, it is consequential.

Reimagining case content to attend to ontological fidelity might mean attending not only to the relevant clinical information, but also to the other human dimensions of a clinical encounter. The patient voice has historically been excluded, in part, due to epistemic injustice: patients are often stereotyped as unreliable sources of information and are therefore denied the capacity to contribute to knowledge generation [48]. The narrative nature of any patient-physician interaction would be made more present by inviting real patients (as well as physicians and other health care providers) to share everyday clinical stories in the case writing process.

We would hear voices—of clinicians, of patients, and of others relevant to the scenario at hand. The words used, the feelings expressed, and the reactions described would be authentic, and the story might not follow a neat or logical timeline—it might even be a bit messy! There may even be room for cases to become progressively messier as learners move through the curriculum and gain skill, knowledge, and confidence, working toward fostering comfort with uncertainty and ambiguity that characterizes human experience, including medical practice.

### Purpose

Despite our best efforts to write cases that lead to rich conversation and inspire deep thinking, the cases that commonly structure our curriculum aim primarily to impart essential information. Consequently, cases often come to serve as a type of checklist.

The ritualized purpose of a case invokes a set of prescribed small-group learning practices that students have come to expect, and—motivated by a workload that is intense and stressful [49]—these are often approached with a ‘let’s get this done’ attitude [2]. Translated into how we approach cases, this means that they are often anticipated to be succinct with limited detail [33]; they simplify complex ideas into easy-to-memorize steps or categories; and they focus on streamlining material that might be assessed on an upcoming exam.

Likewise, cases are expected to unfold in a routine way. Whether negative (ending with the patient’s death, perhaps) or positive (ending with the patient recovering and thriving, for example), learners generally expect the cases to conclude with a concrete resolution. However, if the purpose of cases is to provide a mechanism for students to learn in context, cases ought to simulate the context of real-life medical practice [14,17,30], which is multi-layered and complex [50]. One might expect that cases would be somewhat convoluted, in order to inspire reaction and rich conversation.

If we were to reimagine cases to attend to ontological fidelity, we might reorient cases so that they make space for the stories that constitute medical practice. Cases might present a complicated situation, or one that moves away from the routine and might not be easily resolved. This could foster deeper thinking, introspection, and analysis of the types of challenges they will face moving forward in practice.

## Ontological Fidelity in “Real Life”

Without a doubt, evidence-based scientific and clinical approaches must be present in cases—but these are certainly not the only things that matter. While the integration of a philosophical perspective to cases may seem complicated, in reality, cases already exist as an artefact of our ontological orientation in the field of medicine. Our job is simply to reflect on what we want to represent as real and important.

We believe that, like all education strategies that attempt to simulate future real-life scenarios, cases should attend to the question of fidelity. But, rather than physical or functional fidelity, it is *ontological fidelity* that lies at the heart of every case. Perhaps the simplest way to provide ontological fidelity—to make the cases *feel real* to students—is to base these cases on real people and communicate that to students. However, as any reader of literature or movie-goer can attest, we do not need stories to be true in order for them to feel real. When cases are thoughtfully constructed, they convey universal truths in ways that we recognize to be deeply rooted in reality. In this manner, just as the students in our previous study engaged differently with cadavers compared to manikins because of their realness [12], creating cases that feel authentic may change the way students engage with case-informed learning.

The cases we use for medical education reproduce unquestioned philosophical assumptions about *the nature of reality*. Cases that stick to a prescribed formula help to reinforce a narrow construction of what tutorials and cases should look like and what they can do. As we focus on the idea of ontological fidelity, we encourage educators to broaden their ideas about what cases not only could be, but also what they should be. This means re-examining case format, content, and purpose, but also involves a concerted effort to authentically integrate the “lifeworld,” including story, patient voice, emotion, culture, and experience. We encourage educational administrators and case writers to consider what might be gained by approaching case writing progressively and collaboratively, while attuning to ontological questions relating to the nature of reality. Consulting with, and even inviting real patients, physicians, and other health care providers to share everyday clinical stories in the case writing process would be a good way forward.
